# Premedication with pioglitazone prevents doxorubicin-induced left ventricular dysfunction in mice

**DOI:** 10.1186/s40360-021-00495-w

**Published:** 2021-05-07

**Authors:** Takaaki Furihata, Satoshi Maekawa, Shingo Takada, Naoya Kakutani, Hideo Nambu, Ryosuke Shirakawa, Takashi Yokota, Shintaro Kinugawa

**Affiliations:** 1 Department of Cardiovascular Medicine, Faculty of Medicine and Graduate School of Medicine, Hokkaido University, Kita-15, Nishi-7, Kita-ku, Sapporo, 060-8638 Japan; 2 Faculty of Lifelong Sport, Department of Sports Education, Hokusho University, Ebetsu, 069-8511 Japan; 3 Research Fellow of the Japan Society for the Promotion of Science, Tokyo, Japan

**Keywords:** Anticancer agent, LV dysfunction, Cardiac toxicity, Thiazolidinediones

## Abstract

**Background:**

Doxorubicin (DOX) is widely used as an effective chemotherapeutic agent for cancers; however, DOX induces cardiac toxicity, called DOX-induced cardiomyopathy. Although DOX-induced cardiomyopathy is known to be associated with a high cumulative dose of DOX, the mechanisms of its long-term effects have not been completely elucidated. Pioglitazone (Pio) is presently contraindicated in patients with symptomatic heart failure owing to the side effects. The concept of drug repositioning led us to hypothesize the potential effects of Pio as a premedication before DOX treatment, and to analyze this hypothesis in mice.

**Methods:**

First, for the hyperacute (day 1) and acute (day 7) DOX-induced dysfunction models, mice were fed a standard diet with or without 0.02% (wt/wt) Pio for 5 days before DOX treatment (15 mg/kg body weight [BW] via intraperitoneal [i.p.] administration). The following 3 treatment groups were analyzed: standard diet + vehicle (Vehicle), standard diet + DOX (DOX), and Pio + DOX. Next, for the chronic model (day 35), the mice were administrated DOX once a week for 5 weeks (5 mg/kg BW/week, i.p.).

**Results:**

In the acute phase after DOX treatment, the percent fractional shortening of the left ventricle (LV) was significantly decreased in DOX mice. This cardiac malfunction was improved in Pio + DOX mice. In the chronic phase, we observed that LV function was preserved in Pio + DOX mice.

**Conclusions:**

Our findings may provide a new pathophysiological explanation by which Pio plays a role in the treatment of DOX-induced cardiomyopathy, but the molecular links between Pio and DOX-induced LV dysfunction remain largely elusive.

## Background

Cardiotoxicity, which is generally defined as toxicity that affects the heart, has become a major medical issue, because adverse heart reactions to chemotherapy lead to increased morbidity and mortality [1]. Although the anthracycline antibiotic doxorubicin (DOX) is widely used as a very effective chemotherapeutic drug for hematological as well as solid cancers, DOX unfortunately induces many kinds of cardiac toxic effects, such as transient arrhythmias, nonspecific electrocardiographic abnormalities, and cardiomyopathy [2, 3]. Some clinical studies have suggested that late-onset DOX-induced cardiomyopathy is strongly associated with a high cumulative dose of DOX [4]. For example, doxorubicin leads to a 5% incidence of congestive HF if a cumulative lifetime dose of 400 mg/m^2^ is taken, 26% at 550 mg/m^2^, and 48% at 700 mg/m^2^ [5]. The underlying mechanisms of both acute-onset and late-onset cardiotoxicity have been thought to include several factors, such as oxidative stress, iron overload, mitochondrial dysfunction, and DNA damage [6]. Nonetheless, a standard therapy for the treatment or prevention of DOX-induced cardiomyopathy has not been established to date, which has created an obstacle in cancer treatments.

Thiazolidinediones (TZDs), including pioglitazone (Pio), have been shown to improve the disease state of type II diabetic and metabolic syndrome patients, mainly through the improvement of insulin resistance [7]. More importantly, TZDs were shown to improve left ventricular (LV) function in mice with heart failure (HF) after myocardial infarction [8], and to reduce the risk of major adverse cardiovascular events in people with insulin resistance [9]. Furthermore, there was a study that showed Pio played a protective role in doxorubicin-induced nephropathy in rat to a similar extent as an angiotensin converting enzyme inhibitor, improving profibrotic and inflammatory mechanisms [10]. However, an increased risk of HF owing to fluid retention upon Pio administration was strongly feared [11], such that presently, Pio is contraindicated for patients with symptomatic HF.

In recent years, there has been increasing interest in drug repositioning, which is a concept defined as the process of finding new uses for existing drugs, particularly in the field of cancer therapeutics [12]. Moreover, premedication has been used in other settings to reduce the risk of adverse events [13, 14]. We therefore hypothesized that Pio might reduce the adverse events of DOX, and may improve LV dysfunction caused by DOX treatment if used as a premedication, which is taking Pio before DOX treatment, rather than taking it at the same time or after developing HF. Therefore, to determine whether premedication with Pio prevents DOX-induced LV dysfunction, we analyzed cardiac function in a mouse model of DOX-induced LV dysfunction, with or without Pio premedication.

## Materials and methods

### Ethics statement

The study was conducted in accordance with the ARRIVE guidelines. The Hokkaido University Animal Research Committee approved all experimental procedures and methods of animal care (study approval no.: 13–0074). The Animal Care Guidelines for the Care and Use of Laboratory Animals of Hokkaido University Graduate School of Medicine and the Guide for the Care and Use of Laboratory Animals published by the US National Institutes of Health were applied to all experiments.

### Experimental animals and drug treatment protocol

Eight-week-old male C57BL/6 J mice (23–25 g) were obtained from CLEA Japan, Inc. (Tokyo, Japan). They were maintained on specific diets in a pathogen-free environment in an animal room under controlled conditions on a 12-h light/dark cycle for 5 days. First, the mice were divided into 3 groups. Two of these groups were fed a standard diet (AIN93G, Oriental Yeast Co., Ltd., Tokyo, Japan); the third was fed the standard diet with 0.02% (wt/wt) Pio (pioglitazone hydrochloride; P1901, Tokyo Chemical Industry Co., Ltd., Tokyo, Japan) [15–17]. Next, the group of Pio-pretreated mice and one group of standard diet mice were treated with 15 mg/kg body weight (BW) of DOX (doxorubicin hydrochloride; D1515, Sigma-Aldrich, MO, USA) via intraperitoneal (i.p.) administration [18]. Mice in the other standard diet group were injected with phosphate-buffered saline as the vehicle (Vehicle). For the hyperacute model, 30 mice were divided into 3 groups of 10 mice each; i.e., Vehicle, DOX, and Pio + DOX. For the acute model, 76 mice were divided into 3 groups; i.e., Vehicle (*n* = 25), DOX (*n* = 26), and Pio + DOX (*n* = 26). Furthermore, for the chronic model, mice were treated with an injection of 5 mg/kg BW of DOX via i.p. administration per week for 5 weeks [19], whereas the untreated mice were injected with phosphate-buffered saline as the vehicle. Thirty-five mice were divided into 3 groups; i.e., Vehicle (*n* = 7), DOX (*n* = 14), and Pio + DOX (*n* = 14) [18, 20–22]. One day and seven days after DOX treatment, or 5 weeks after the beginning of weekly injections, the hearts were excised after mice were euthanized under deep anesthesia with 0.3 mg/kg BW of medetomidine (Dorbene® Kyoritsuseiyaku Co., Ltd., Tokyo, Japan), 4.0 mg/kg BW of midazolam (Dormicum®, Astellas Pharma Inc., Tokyo, Japan), and 5.0 mg/kg BW of butorphanol (Vetorphale®, Meiji Seika Kaisha, Ltd., Tokyo, Japan) (MMB mixture) [23].

### Survival analysis

Survival was analyzed in all groups of acute DOX model mice, i.e., Vehicle (*n* = 15), DOX (*n* = 15), and Pio + DOX (*n* = 15). During the 7 days after DOX injection, the cages were inspected daily for dead animals.

### Echocardiographic measurements

Echocardiographic measurements were performed both 1 day and 7 days after drug treatments, under light anesthesia with the MMB mixture. Two-dimensional parasternal short-axis views were obtained at the levels of the papillary muscles. On-axis, two-dimensional targeted M-mode tracings, for at least 20 cardiac cycles, were electronically recorded (Aplio 300, TOSHIBA, Tokyo, Japan; EUB-8000, HITACHI, Tokyo, Japan). The following indexes were analyzed using the software in the echo instrument; LV end-diastolic diameter (LVEDD), LV end-systolic diameter (LVESD), percent fractional shortening (%FS), heart rates (HR), and LV wall thickness [23–28].

### Histological analyses

Hearts were excised, fixed in 4% paraformaldehyde, embedded in paraffin, cut into three transverse sections (apex, middle ring, and base), and stained with HE for histological analyses. Morphological analysis of the cross-sectional area per cell was performed in at least 20 cells from each mouse, chosen appropriate cross-section and in the vertically aligned muscle layers adjacent to them, not tangentially [29, 30]. For evaluation of the degree of fibrosis, hearts were stained with MT. After collagen fiber identification and quantification, collagen fiber areas were measured with ImageJ software (NIH, MD, USA) [31]. Pictures were acquired using a microscope (BZ-X710, Keyence, Osaka, Japan) [23, 32].

### Quantitative reverse transcription polymerase chain reaction (PCR)

Total RNA was extracted from heart tissues with QuickGene-810 (FujiFilm, Tokyo, Japan) according to the manufacturer’s instructions. Total RNA concentration and purity were analyzed by measuring the optical density (230, 260, and 280 nm) with a Nanodrop 1000 spectrophotometer (Thermo Fisher Scientific, MA, USA). cDNA was synthesized with a high capacity cDNA reverse transcription kit (Applied Biosystems, CA, USA). Reverse transcription was performed for 10 min at 25 °C, 120 min at 37 °C, 5 s at 85 °C, and then the solution was cooled at 4 °C. TaqMan quantitative real-time PCR was performed with the 7300 real-time PCR system (Applied Biosystems) to amplify interleukin-1beta (*Il1b*) (Mm00434227_g1) and tumor necrosis factor-alpha (*Tnfa*) (Mm01161290_g1) cDNA in the heart. After 2 min at 50 °C and 10 min at 95 °C, PCR amplification was performed for 40 cycles of 15 s at 95 °C and 1 min at 60 °C. Glyceraldehyde-3-phosphate dehydrogenase (GAPDH) was used as an internal control. Data were analyzed using the comparative 2^-ΔΔCT^ method [25, 26, 33–35].

### Preparation of isolated mitochondria

Heart tissues were quickly harvested after the mice were sacrificed, and mitochondria were isolated as previously described, and their protein concentrations were measured as described [25, 36]. Briefly, after incubation for 2 min in mitochondrial isolation buffer (100 mmol/L sucrose, 100 mmol/L KCL, 1 mmol/L KH_2_PO_4_, 0.1 mmol/L ethyleneglycol bis (2-aminoethyl ether)-N,N,N′,N′ tetraacetic acid (EGTA), 0.2% bovine serum albumin, and 50 mmol/L Tris-HCl; pH 7.4) containing 0.1 mg/mL proteinase (P8038, Sigma-Aldrich, MO, USA), minced hearts were homogenized with a motor-driven Teflon pestle in a glass chamber. Homogenates were centrifuged at 700 *g* for 10 min, and then the supernatants were centrifuged at 10,000 *g* for 10 min. Pellets were suspended in suspension buffer (225 mmol/L mannitol, 75 mmol/L sucrose, 10 mmol/L Tris, and 0.1 mmol/L ethylenediaminetetraacetic acid [EDTA], pH 7.4) [25].

### Mitochondrial oxidative phosphorylation (OXPHOS) capacity and reactive oxygen species (ROS) production with non-fatty-acids or fatty-acids in isolated heart mitochondria

The mitochondrial respiratory capacity from non-fatty-acids were measured in isolated heart mitochondria using a high-resolution respirometer (Oxygraph-2 k, Oroboros Instruments, Innsbruck, Austria), as described previously [25, 26, 37]. The following two protocols were used for the non-fatty-acid substrates and fatty-acid substrates. After the addition of isolated mitochondria of LV to the chamber in the respirometer filled with 2 mL of MiR05 (in mmol/L: sucrose 110, K-lactobionate 60, EGTA 0.5, MgCl_2_ 3, taurine 20, KH_2_PO_4_ 10, 4-(2-hydroxyethyl)-piperazineethanesulfonic acid [HEPES] 20, 1% bovine serum albumin [BSA], pH 7.1), we added the substrates, and adenosine diphosphate (ADP) in the following order.

Protocol 1 (non-fatty-acid substrates):

- (1) malate 2 mmol/L + pyruvate 10 mmol/L (complex I-linked substrates), (2) glutamate 10 mmol/L (a complex I-linked substrate), (3) ADP (10 mmol/L), and (4) succinate 10 mmol/L (a complex II-linked substrate).

Protocol 1 (non-fatty-acid substrates):

- (1) malate 2 mmol/L, (2) octanoyl-l-carnitine 0.15 mmol/L, and (3) ADP 10 mmol/L.

The respiratory rates (i.e., O_2_ consumption rates) were expressed as the O_2_ flux normalized to the mitochondrial protein concentration. Datlab software (Oroboros Instruments) was used for data acquisition and data analysis [25, 26, 38].

Mitochondrial ROS generation simultaneously were measured with the mitochondrial respiratory capacity in the isolated mitochondria of LV using a spectrofluorometer (Fluorescence LED2-Module; Oroboros Instruments, Innsbruck, Austria) equipped with a respirometer, as previously described [39, 40]. Mitochondrial ROS were evaluated after the conversion of mitochondrial superoxide (O^2−^) into H_2_O_2_ by the addition of superoxide dismutase (SOD). SOD (5 U/ mL), horseradish peroxidase (1 U/mL), and Ample® UltraRed reagent (10 μmol/L; Thermo Fisher Scientific, Waltham, MA) were added to the chamber of the respirometer. H_2_O_2_ reacts with Amplex UltraRed in a 1:1 stoichiometry catalyzed by horseradish peroxidase, which yields the fluorescent compound resorufin. The excitation wavelength (525 nm) and fluorescence detection (587 nm) were selected. The fluorescence of resorufin was continuously monitored during the protocol 1 (along with the measurements of mitochondrial respiratory capacity). In order to eliminate the possible interference of substrates, the H_2_O_2_ generation rate was calibrated by the titration of H_2_O_2_ in 0.1 μmol/L increments before and after each substrate addition [39, 40].

### Measurement of mitochondrial iron contents

After collecting isolated mitochondria using Mitochondrial Isolation Kit for Tissue (Pierce, MD, USA), mitochondrial iron contents were measured using a commercial Iron Assay Kit (BioAssay Systems, CA, USA) according to the manufacturer’s protocol [41].

### Statistical analysis

Statistical analyses were performed by commercially available software, GraphPad Prism, version 7.05 (GraphPad Software, CA, USA). Data are expressed as means ± standard error (SE). Two-way analysis of variance (ANOVA) was used to analyze the effects of drug, time, and interaction. The Sidak test was performed for multiple-group comparisons of the means at each time point. One-way ANOVA was used to analyze the effects of the drug, followed by the Tukey test for multiple-group comparisons of means. *P*-values of less than 0.05 were considered to indicate a statistically significant difference between 2 groups.

## Results

### Premedication with Pio prevents LV dysfunction caused by DOX treatment in the acute phase (day 7), but not in the hyperacute phase (day 1)

To analyze the effects of LV function after the administration of DOX and Pio, we first analyzed 2 groups, i.e., standard diet + vehicle (Vehicle) and Pio + vehicle (Pio), on LV function as a preliminary study. There was no significant difference in %FS, which is known as one of the simplest ways to assess LV function [23–25, 27, 42–44], between the 2 groups at 7 days after drug treatment (Vehicle: 55.3% ± 2.4% vs. Pio: 53.5% ± 1.8%, *n* = 10 each). Therefore, we performed the subsequent experiments on all mice except for the Pio group, i.e., the Vehicle, standard diet + DOX (DOX), and Pio + DOX groups.

**Fig. 1:**
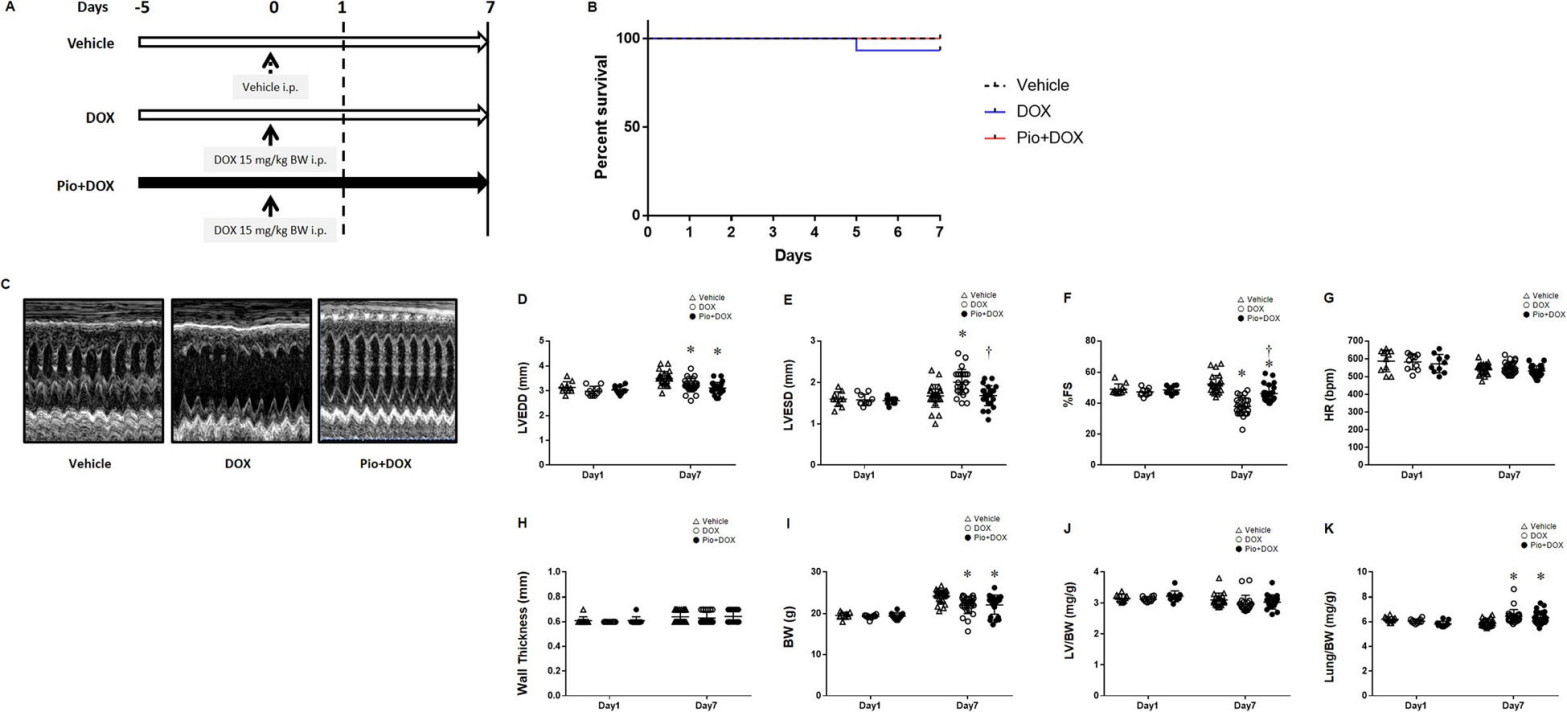
Pio pretreatment partially prevented DOX-induced LV dysfunction in the acute phase (day 7), but not in the hyperacute phase (day 1). **a** Diagram showing the treatment course of each group. For the hyperacute and acute phases, mice were divided into 3 groups. Vehicle mice were fed a standard diet and treated with vehicle instead of DOX. DOX mice were fed a standard diet and treated with a single DOX bolus (15 mg/kg of body weight [BW]) on day 0. Pio + DOX mice were orally administered with Pio (0.02% [wt/wt]) from day − 5 as premedication, and then treated with a DOX bolus on day 0. All measurements were acquired and hearts were excised on both day 1 (hyperacute phase) and day 7 (acute phase). The horizontal open arrows represent standard diet intake, and the horizontal closed arrow indicates Pio intake. The vertical arrows indicate treatment with vehicle or DOX. **b** Kaplan-Meier survival curves of Vehicle (*n* = 15, black broken line), DOX (*n* = 15, blue line), or Pio + DOX mice (*n* = 15, red line). **c** Representative echocardiographs of Vehicle (*left* panel), DOX (middle panel), and Pio + DOX (*right* panel) mice. **d**, **e**, **f**, **g**, **h** Summary data of LVEDD, LVESD, %FS, HR, and LV wall thickness. Day 1: Vehicle (open upward triangles, *n* = 10), DOX (open circles, *n* = 10), and Pio + DOX (closed circles, *n* = 10). Day 7: Vehicle (*n* = 25), DOX (*n* = 26), and Pio + DOX (*n* = 25). **i**, **j**, **k** Summary data of BW, LV/BW, and lung/BW. Data are shown as means ± SEs. *P < 0.05 vs. Vehicle, †*P* < 0.05 vs. DOX. DOX, doxorubicin; Pio, pioglitazone; LV, left ventricle; LVEDD, LV end-diastolic diameter, LVESD, LV end-systolic diameter; %FS, percent fractional shortening

In the protocol for the hyperacute and acute phases (Fig. 1a), only one mouse in the DOX group died, and no other mice died regardless of DOX or Pio treatment (Fig. 1b). LV function was evaluated by echocardiography at both 1 day and 7 days after DOX treatment via i.p. administration. In the hyperacute phase on day 1, LV dysfunction was not yet detectable. However, in the acute phase on day 7 (Fig. 1c), DOX treatment caused LVEDD to be lower than that in vehicle-treated mice, regardless of a standard diet or Pio intake (Fig. 1d). Interestingly, LVESD in the DOX mice was higher than that in the Vehicle mice, even though LVESD in the Pio + DOX mice was comparable to that of Vehicle mice (Fig. 1e). %FS in the DOX mice and Pio + DOX mice was decreased compared with that in Vehicle mice, and that in Pio + DOX mice was increased compared with that in DOX mice (Fig. 1f). Thus, DOX treatment induced an impairment in LV contractile function, whereas Pio treatment preserved LV contractile function to some extent, suggesting that premedication with Pio partially protected LV function in the acute phase of DOX-induced LV dysfunction. HR during the measurement of echocardiography did not differ among the 3 groups (Fig. 1g). Likewise, echocardiography displayed no differences in LV wall thickness among the 3 groups (Fig. 1h).

### DOX treatment decreased the body weight (BW) of mice in the acute phase (day 7)

In the hyperacute phase on day 1, no differences were observed among the groups in BW, LV/BW, and lung/BW. In the acute phase on day 7, BW in DOX mice and Pio + DOX mice were lower than that in Vehicle mice, i.e., BW were lower in DOX-treatment mice than in vehicle-treated mice (Fig. 1i). The possible reason as to why BW in DOX-treated mice were decreased is the side effects of DOX itself, which include weight loss owing to the decrease in food intake after DOX administration. LV/BW did not differ among the groups (Fig. 1j). Lung/BW in DOX mice and Pio + DOX mice was increased compared with that in Vehicle mice (Fig. 1k). This change did not appear to be caused by increased lung weight, which might imply a state of slight lung congestion caused by HF, but may rather be a result of decreased BW in these treatment groups.

### Pio inhibits vacuolization in cardiomyocytes treated with DOX

**Fig. 2:**
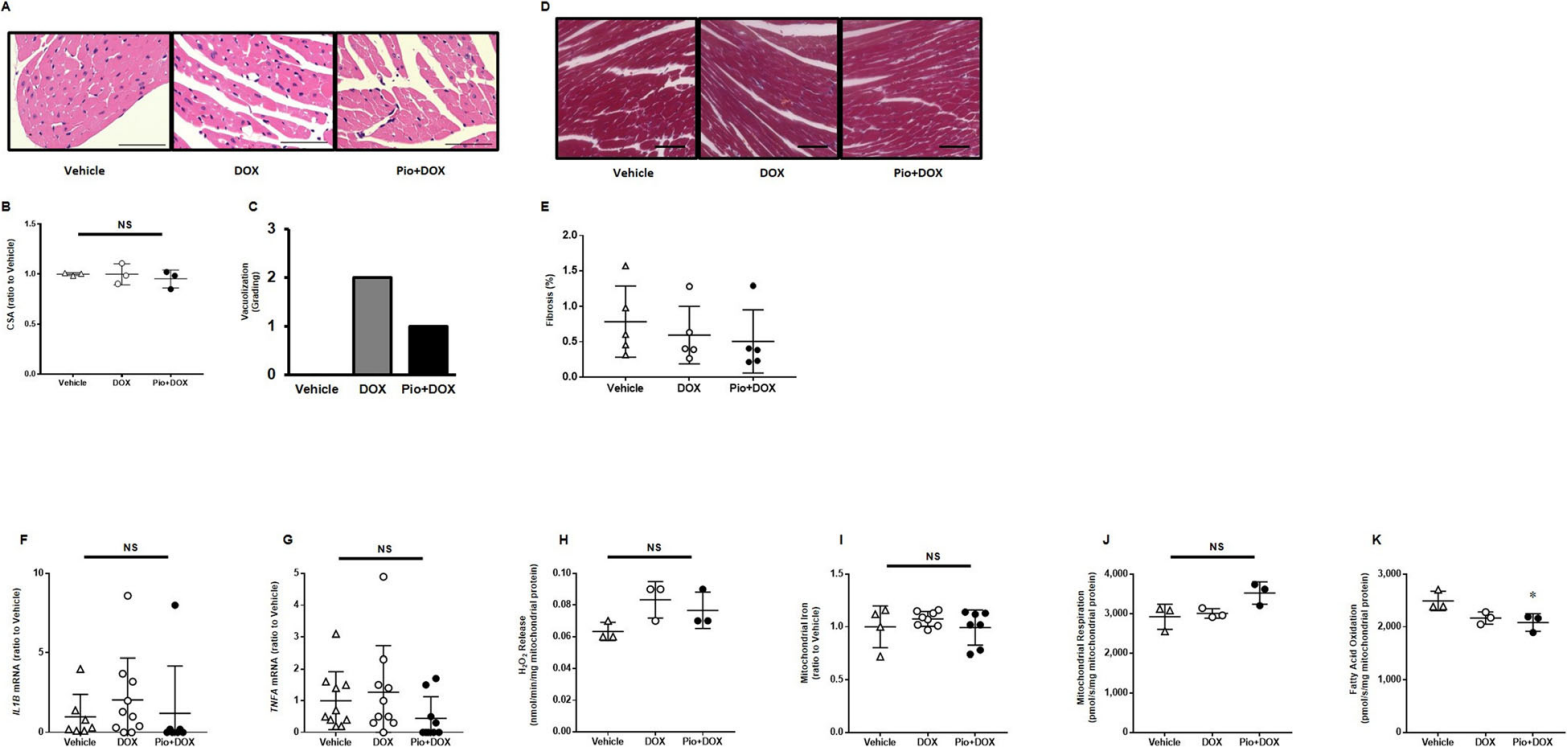
Pio improved vacuolization but showed no significant biochemical or physiological changes in the acute phase of DOX-induced LV dysfunction mice (day 7). **a** Representative images of hematoxylin-eosin (HE) staining of a heart from each group. Scale bar, 50 μm. **b** Summary data of the cross-sectional area (CSA) of the left ventricle on HE staining. *n* = 3 for each group. **c** Number of vacuolization in cells in the HE stained samples of each sample, calculated as the percentage of cells containing vacuoles, i.e., 0: none; 1: less than 25%; 2: 25–50%; and 3: more than 50%. **d** Representative images of Masson trichrome (MT) staining of a heart from each group. Scale bar, 50 μm. **e** Summary data of fibrosis area in the MT-stained samples. *n* = 3 for each group. **f**, **g** Quantitative analysis of the gene expression levels of interleukin-1beta (*Il1b*) and tumor necrosis factor-alpha (*Tnfa*) in the heart on day 7 (*n* = 7–10 for each group). **h** Hydrogen peroxide (H_2_O_2_) release originating from non-fatty-acid substrates from isolated mitochondria in hearts during state 3 respiration (i.e. oxidative phosphorylation) using the complex I- and II-linked substrates glutamate, malate, and succinate (*n* = 3 for each group). **i** Levels of mitochondrial iron contents (*n* = 4–8 for each group). **j** Mitochondrial respiration using non-fatty-acid substrates in isolated mitochondria from the heart during state 3 respiration using the complex I- and II-linked substrates glutamate, malate, and succinate (*n* = 3 for each group). **k** Mitochondrial respiration with fatty-acid substrate (octanoyl-l-carnitine) in isolated mitochondria from the heart during state 3 respiration (*n* = 3 for each group). Data are shown as means ± SEs. *P < 0.05 vs. Vehicle. NS, not significant

Histological analysis with hematoxylin-eosin (HE) showed that myocyte cross-sectional areas did not differ among the 3 groups (Fig. 2a, b). These results suggested that myocyte cellular did not show hypertrophy or atrophy due to DOX and Pio treatment. The number of vacuolization in cells in DOX and Pio + DOX mice was higher than that in Vehicle mice, whereas that in Pio + DOX mice was lower than that in DOX mice (Fig. 2c). As assessed by Masson trichrome (MT) staining, the degree of myocardial fibrosis did not differ among the 3 groups (Fig. 2d, e).

### No significant mechanistic changes occurred in the heart in the acute phase (day 7)

To clarify the underlying mechanism of DOX-induced acute LV dysfunction, we first measured the levels of proinflammatory cytokines. Gene expression levels of interleukin-1beta (*Il1b*) and tumor necrosis factor-alpha (*Tnfa*) were not significantly increased in DOX-treated mice (Fig. 2f, g). Next, there was also no significant difference in mitochondrial reactive oxygen species (ROS), i.e., hydrogen peroxide (H_2_O_2_) release rates from isolated mitochondria during mitochondrial respiration (Fig. 2h). In accordance with ROS production, the iron content in mitochondria did not differ among the groups (Fig. 2i). We measured mitochondrial state 3 respiration using glutamate, malate, and succinate as the complex I- and II-linked substrates, and found comparable results among the groups (Fig. 2j). We also measured mitochondrial state 3 respiration using malate and octanoyl-l-carnitine, i.e., fatty acid oxidation (FAO). FAO in Pio + DOX mice was lower than that in Vehicle mice, whereas FAO in DOX and Pio + DOX did not differ between the groups (Fig. 2k).

### Premedication with Pio preserves LV function after DOX treatment in the chronic phase (day 35)

**Fig. 3:**
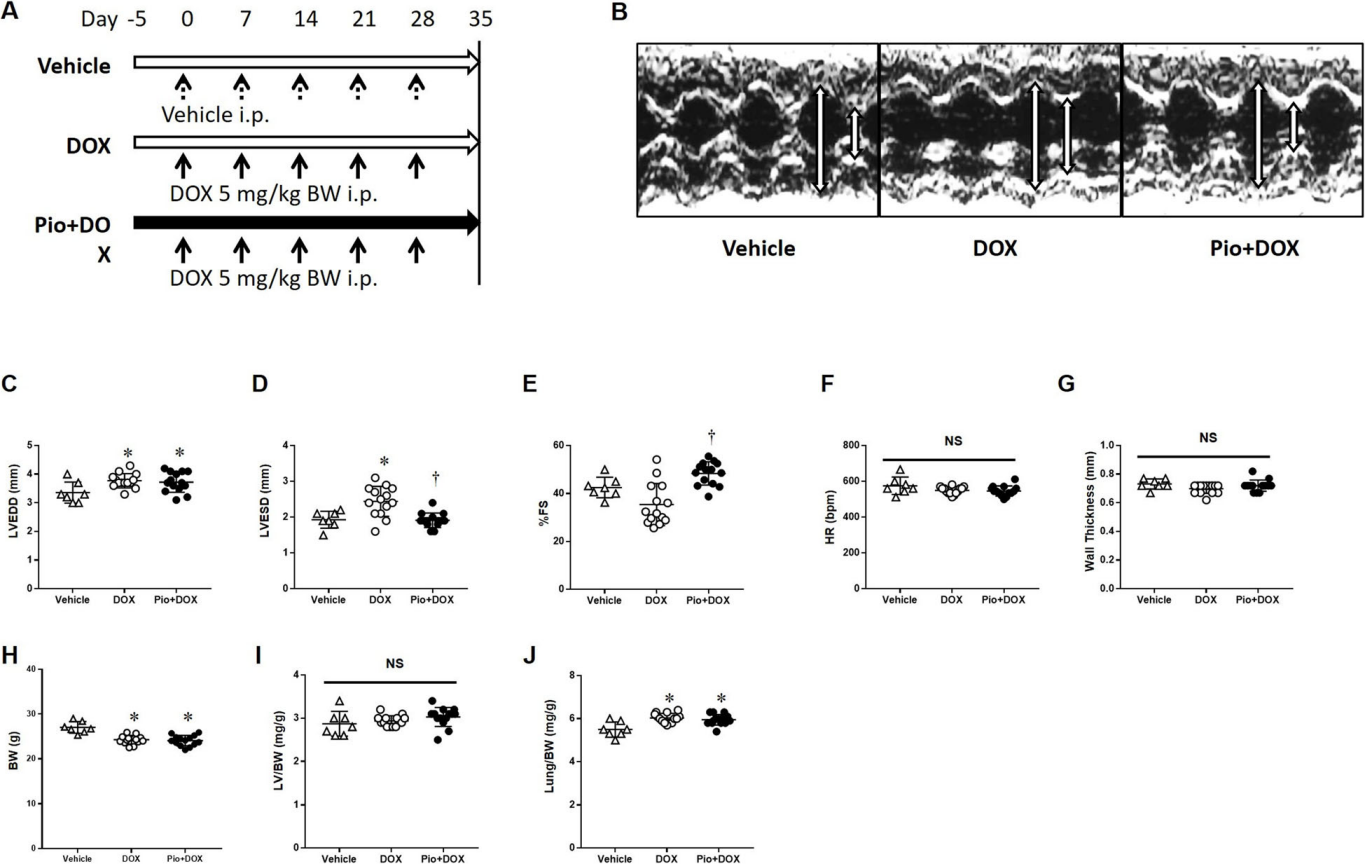
Pio pretreatment prevented LV dysfunction in the chronic phase of DOX-induced LV dysfunction mice (35 days). **a** Diagram showing the treatment course of each group. For the chronic phase, mice were divided into 3 groups. Vehicle mice were fed a standard diet and treated with vehicle instead of DOX. DOX mice were fed a standard diet and treated with weekly DOX injections for 35 days (25 mg/kg cumulative dose). Pio + DOX mice were orally administered with Pio (0.02% [wt/wt]) from day − 5 as premedication and were treated with weekly DOX injections for 35 days. Measurements of BW and food intake were performed every week. Cardiac function was evaluated by echocardiography and hearts were excised on day 35. Open horizontal arrows represent standard diet intake, and the closed horizontal arrow indicates Pio intake. The vertical arrows indicate treatment with vehicle or DOX. **b** Representative echocardiographs from a mouse in each group; Vehicle *(left* panel), DOX (*middle* panel), and Pio + DOX (*right* panel). The vertical arrows in the echocardiograph indicate LVEDD (*left* arrow) and LVESD (*right* arrow). **c**, **d**, **e**, **f**, **g** Summary data of LVEDD, LVESD, %FS, HR, and LV wall thickness. Vehicle (open upward triangles, *n* = 7), DOX (open circles *n* = 14), and Pio + DOX (closed circles, *n* = 14). **h**, **i**, **j** Summary data of BW, LV/BW, and lung/BW (*n* = 7–14 for each group). Data are shown as means ± SEs. *P < 0.05 vs. Vehicle; †*P* < 0.05 vs. DOX

To confirm the effects of DOX and Pio in the chronic phase, we used a chronic model of DOX treatment, as shown in Fig. 3a. In this protocol for the chronic phase, DOX administration appeared to affect the amount of food intake (Table 1), even though no mice died in any group regardless of DOX or Pio treatment. LVEDD in both DOX and Pio + DOX mice was lower than that in Vehicle mice (Fig. 3b, c). LVESD in the DOX mice was higher than that in Vehicle mice, whereas LVESD in the Pio + DOX mice was comparable to that of Vehicle mice (Fig. 3d). %FS in the DOX mice had a tendency to be lower than that of Vehicle mice, and that in Pio + DOX mice was substantially increased compared with that in DOX mice (Fig. 3e). Importantly, these patterns of LV function on day 35 were quite similar to those of day 7. HR and LV wall thickness did not differ among the 3 groups (Fig. 3f, g). BW in DOX mice and Pio + DOX mice was decreased compared with that in Vehicle mice (Fig. 3h). LV/BW did not differ among the groups (Fig. 3i), even though lung/BW in DOX mice and Pio + DOX mice was increased compared with that in Vehicle mice (Fig. 3j).

**Table 1:** Body weights and food intake of chronic DOX-induced cardiomyopathy model mice during 35 days

*n*	Vehicle	DOX	Pio + DOX
	10	20	20
**Body weights (g)**			
Day 0	24.9 ± 0.5	26.3 ± 0.3*	26.6 ± 0.2*
Day 7	25.5 ± 0.5	25.4 ± 0.3	25.7 ± 0.2
Day 14	26.1 ± 0.5	25.4 ± 0.3	25.1 ± 0.2
Day 21	26.7 ± 0.5	24.6 ± 0.3*	24.2* ± 0.2*
Day 28	27.1 ± 0.5	24.4 ± 0.2*	23.9* ± 0.3*
Day 35	27.4 ± 0.5	23.9 ± 0.2*	23.4* ± 0.3*
**Food intake (g/day)**			
Day 0	2.6	3.1	3.4
Day 7	2.7	2.1	2.1
Day 14	2.8	2.5	2.4
Day 21	2.7	2.2	2.1
Day 28	2.6	2.2	2.0
Day 35	2.5	2.0	1.9

Data are shown as the mean ± SEs (body weights) or mean (food intake). *P < 0.05 vs. Vehicle, *n* Number, *DOX* Doxorubicin, *Pio* Pioglitazone

## Discussion

This is the first report to demonstrate that premedication with Pio partially prevents LV dysfunction in both the acute and chronic phases, but not in the hyperacute phase, in mice treated with DOX, suggesting that the duration from DOX administration could be crucial for the emergence of the DOX-induced cardiomyopathy. Premedication with Pio may be a novel therapeutic strategy for the prevention of DOX-induced cardiomyopathy. However, molecular links between Pio and DOX-induced LV dysfunction remain largely elusive.

In terms of LV function, Pio treated mice without DOX treatment did not differ from vehicle mice on preliminary examination. This echocardiographic evaluation supports the idea that the effects of Pio on LV function are limited in the nondisease condition.

In addition to day 7, we also performed measurements on day 1, because we assumed that differences in the effects of premedication and treatment after DOX administration would be found at an early stage. Similar to our results, there were no cardiomyopathy phenotypes (e.g., LV dysfunction and LV/BW) in the hyperacute phase until day 1 after DOX treatment [45–47]. Initially, chemotherapeutic agents do not appear to affect DOX-induced cardiomyopathy, which was also the case in our present study. Therefore, these results suggest that the duration from DOX administration, as well as the cumulative dose of DOX, could be crucial for the emergence of the DOX-induced cardiomyopathy. However, several signaling pathways, including cell death, were found to be altered during the hyperacute phase immediately after DOX treatment [45–47]. In fact, various studies demonstrated that drugs providing protective effects against DOX-induced acute cardiomyopathy were often administered before DOX treatment, and their effects might depend on the mechanism of the drug or the animal model [48–50]. Saraogi et al. observed that LV dysfunction was worse in rats treated with Pio (10 mg/kg BW) for 14 days and with DOX injection (15 mg/kg BW, single. dose) on the tenth day, than in rats treated with Pio only [51]. These results, which are the opposite of our results, were induced partially because the model used in their experiment was different from ours (animal, drug dose, and drug protocol). As there was no physiological evaluation in this previous study, judging from the side effects and oxidative stress data, we estimate that rat hearts were affected much more by DOX treatment than our mouse hearts. We believe that the extent of the DOX-induced heart damage occurring during the acute phase may determine whether Pio plays a protective or an aggravating role in DOX-induced LV dysfunction.

A previous report showed that an increase in LV end-systolic volume and a tendency of LV end-diastolic volume to decrease in DOX-treated mice were found in the acute phase after an administration of DOX (20 mg/kg BW single i.p.) [52]. Moreover, mouse models of DOX treatment vary, even in their i.p. administration; for example, a 10 mg/kg BW single bolus (acute protocol), or 5 injections of 4 mg/kg BW/week (20 mg/kg cumulative dose, chronic protocol) [53]. In the present study, for the acute model, we first used a 15 mg/kg BW bolus injection via i.p. administration, because a simple protocol, i.e., a single injection, appeared to be preferable for determining the acute effects of DOX treatment. In addition, a much higher single-injection dose of 20 mg/kg BW or more readily caused mice to die in our preliminary experiments. Multiple lower-dose injections might be a more favorable choice, because a single-dose protocol makes it difficult to understand how DOX works in this situation. As cancer patients are usually treated with multiple low doses to limit adverse events, including cardiotoxicity, other protocols may also be useful, such as different doses and timings of injection. We next added mice receiving one injection of 5 mg/kg BW of DOX via i.p. administration per week for 5 weeks as the chronic model, and our results suggested that Pio may have protective effects on both acute and chronic DOX-induced LV dysfunction. From the point of view of clinical application, it is important to clarify whether the Pio concentrations achieved in mice can also be achieved in actual cancer patients. Moreover, such additional studies are expected to lead to the clarification of the mechanism of premedication with Pio, not only regarding pathology of the acute phase but also regarding pathology of the chronic phase.

Although Pio can alter metabolic status to some extent, it also has side effects. In fact, Pio has been used in various doses in rodents, from 0.005 to 0.1%, but most often from 0.01 to 0.05% [15–17]. In addition, different doses might lead to different results. However, particularly in terms of reducing the side effects of Pio, such as fluid retention, which is a serious adverse reaction, 0.02% (wt/wt) of Pio appears to be a reasonable dose for rodents.

The models of DOX-induced cardiomyopathy or LV dysfunction with the heart in rodents have been well characterized with histopathology, serum markers, molecular pathways analysis. But we were unable to clarify the mechanism involved in our study, even though we analyzed inflammation, ROS production, and lipid metabolism. A previous paper proposed that PPARγ upregulation limits DOX-induced cellular toxicity [54]. This new perspective could give us supportive evidence with the phenomenon to the current study.

To date, no effective drug has been established against DOX-induced cardiomyopathy, even though several drugs, such as dexrazoxane, carvedilol, flavonoids, probucol, sildenafil, etc., are thought to be candidates [55]. Pio is well known to carry risks for symptomatic HF patients [11]. Accordingly, to date, no cardiologist has attempted the use of Pio for DOX-induced cardiomyopathy in clinical situations. However, with the recent increased interest in drug repositioning, defined as the process of finding new uses for existing drugs, we considered Pio as a candidate drug for the prevention of DOX-induced cardiomyopathy. Because premedication with Pio is used for patients with normal LV function, this usage does not compete with the idea that Pio is not recommended in patients with symptomatic HF. Unlike sudden-onset diseases, such as myocardial infarction and incipient heart failure, chemotherapy is a scheduled insult to the heart, and hence premedication with Pio may be reasonable from the perspective of the prevention of DOX-induced cardiomyopathy.

## Conclusions

In conclusion, our findings may provide a new pathophysiological explanation for the therapeutic effects of Pio on DOX-induced cardiomyopathy; however, the molecular link between Pio and DOX-induced LV dysfunction remain largely elusive.

## Data Availability

The datasets generated during or analyzed during the present study are available from the corresponding author upon reasonable request.

## Abbreviations

ANOVA: Analysis of variance
BW: Body weight
DOX: Doxorubicin
HF: Heart failure
HR: Heart rates
i.p.: Intraperitoneal
LV: Left ventricle
LVEDD: LV end-diastolic diameter
LVESD: LV end-systolic diameter
%FS: Percent fractional shortening
Pio: Pioglitazone
TZDs: Thiazolidinediones
SE: Standard error
